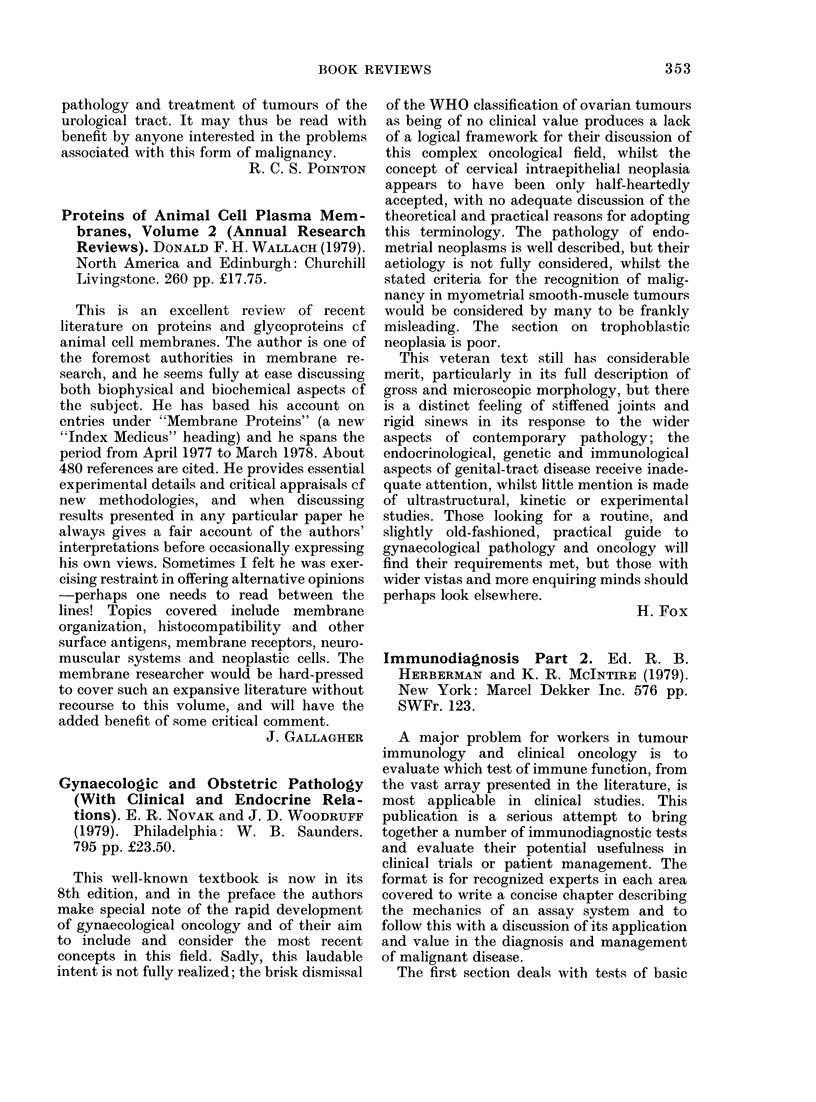# Gynaecologic and Obstetric Pathology (With Clinical and Endocrine Relations)

**Published:** 1980-08

**Authors:** H. Fox


**Gynaecologic and Obstetric Pathology (With Clinical and Endocrine Relations).** E. R. Novak and J. D. Woodruff (1979). Philadelphia: W. B. Saunders. 795 pp. £23.50.

This well-known textbook is now in its 8th edition, and in the preface the authors make special note of the rapid development of gynaecological oncology and of their aim to include and consider the most recent concepts in this field. Sadly, this laudable intent is not fully realized; the brisk dismissal of the WHO classification of ovarian tumours as being of no clinical value produces a lack of a logical framework for their discussion of this complex oncological field, whilst the concept of cervical intraepithelial neoplasia appears to have been only half-heartedly accepted, with no adequate discussion of the theoretical and practical reasons for adopting this terminology. The pathology of endometrial neoplasms is well described, but their aetiology is not fully considered, whilst the stated criteria for the recognition of malignancy in myometrial smooth-muscle tumours would be considered by many to be frankly misleading. The section on trophoblastic neoplasia is poor.

This veteran text still has considerable merit, particularly in its full description of gross and microscopic morphology, but there is a distinct feeling of stiffened joints and rigid sinews in its response to the wider aspects of contemporary pathology; the endocrinological, genetic and immunological aspects of genital-tract disease receive inadequate attention, whilst little mention is made of ultrastructural, kinetic or experimental studies. Those looking for a routine, and slightly old-fashioned, practical guide to gynaecological pathology and oncology will find their requirements met, but those with wider vistas and more enquiring minds should perhaps look elsewhere.

H. Fox